# Psychosocial Adjustment as a Mediator in the Relationship between Childhood Exposure to Maternal Depression and Subsequent BMI and Overweight Risk

**DOI:** 10.3390/children11040441

**Published:** 2024-04-06

**Authors:** Lijie Niu, Skylar Hanson, Juanita Preciado-Becerra, Abdulaziz Eskandarani, Xiaomeng Lei, Mi Le, Zhongzheng Niu, Bin Xie

**Affiliations:** 1 School of Community and Global Health, Claremont Graduate University, Claremont, CA 91711, USA; lijie.niu@cgu.edu (L.N.); shanson@phi.org (S.H.); juanita.preciado@cgu.edu (J.P.-B.); aeskandarani@moh.gov.sa (A.E.); xiaomeng.lei@cgu.edu (X.L.); mi.le@cgu.edu (M.L.); zhongzheng.niu@alumni.cgu.edu (Z.N.); 2 Department of Radiology and Neurology, Keck School of Medicine, University of Southern California, Los Angeles, CA 90033, USA; 3 Department of Population and Public Health Sciences, Keck School of Medicine, University of Southern California, Los Angeles, CA 90033, USA

**Keywords:** BMI, obesity, psychosocial adjustment, maternal depression

## Abstract

Objectives: This study investigated the correlation between early exposure to maternal depression (from 1 month to Grade 3) and the body mass index (BMI) and potential for overweight in adolescents at age 15. It further examined if the pathway of this correlation was influenced by psychosocial adjustment during mid-childhood (Grade 3 to Grade 6), specifically through internalizing and externalizing behaviors. Methods: Our study utilized data from 844 participants in the NICHD Study of Early Child Care and Youth Development (SECCYD) to assess the effects of maternal depression, observed from when the children were one month old to Grade 3, on BMI and the likelihood of overweight or obesity in adolescents aged 15. We also explored whether the average scores of internalizing and externalizing behaviors between Grades 3 and 6 mediated the impact of early maternal depressive symptoms on subsequent health outcomes. The analysis was adjusted for demographic and socioeconomic factors. Results: Findings revealed that internalizing and externalizing behavioral issues significantly mediated the relationship between prolonged maternal depression exposure and subsequent BMI, as well as the risk of overweight or obesity, in adolescents at age 15. Notably, this mediating effect was predominantly evident in girls. Conclusions: Our research demonstrated that the correlation between prolonged exposure to maternal depressive symptoms in childhood and increased BMI and overweight risk in adolescence was significantly mediated through psychosocial adjustment behaviors. We advocate for further exploration of additional mediating factors in future studies.

## 1. Introduction

Over the past forty years, there has been a dramatic escalation in obesity among the youth, with numbers increasing by more than 100 million worldwide [1]. The issue of obesity among the young population has also seen a notable rise within the United States (U.S.). Specifically, around 1 in 5 children are obese [2]. Childhood obesity has emerged as a critical global health challenge, due to its potential to lead to adverse mental and physical health outcomes. This condition often extends into adulthood, increasing the risk for various metabolic disorders, such as type 2 diabetes, cardiovascular diseases, and elevated triglyceride levels, along with mental health concerns, including depression, and emotional and behavioral disorders [3,4]. The factors contributing to childhood obesity are multifaceted, encompassing genetic, metabolic, and environmental factors. Importantly, parenting styles, particularly maternal psychopathology, family dynamics, and coping mechanisms, play a crucial role in shaping children’s interactions with obesogenic environments [4]. Mothers significantly influence their children’s lifestyle choices, affecting diet and activity levels, thereby impacting weight development. Besides, peer support during childhood and adolescence has been identified as influential in affecting weight control strategies and behaviors, with a lack of support linked to poor psychosocial outcomes and higher levels of depression, loneliness, and anxiety [5]. Moreover, adverse childhood experiences and short sleep durations have been recognized as contributing factors to obesity [1]. Given the complex interplay of environmental conditions and psychosocial adjustment in the risk of becoming overweight, it is imperative to explore multifaceted intervention strategies that address the underlying genetic, environmental, and psychosocial contributors to childhood obesity.

Postpartum depression is a significant problem in the U.S., affecting 10–15% of women [6], with potential long-term consequences extending into later life. The relationship between maternal postpartum depression and obesity in children is increasingly recognized. Studies suggest this relationship may be connected to depression’s impact on parenting quality, leading to insufficient care, communication, and responsiveness [7,8]. Maternal depression can impair children’s cognitive, emotional, and behavioral development [9]. Child behavioral issues are classified into internalizing disorders, including anxiety and depression, and externalizing disorders, such as aggression and delinquency [10]. Studies indicate that these behavioral disorders, characterized by negative mood and behavioral inhibition, respectively, are significantly influenced by maternal depression [11]. This influence can contribute to unhealthy eating habits, reduced physical activity, and increased sedentary behaviors among children, which are recognized as primary contributors to the risk of obesity [12,13,14]. Recognizing the relationship between maternal mental health and child behavior is essential for developing effective interventions aimed at mitigating childhood obesity risks. By focusing on integrated strategies that target both maternal well-being and child health behaviors, we can better address the multifaceted nature of childhood obesity. Therefore, understanding the role of child behavior as a mediating factor in the relationship between maternal depression and childhood obesity is crucial for developing effective, comprehensive interventions.

Despite the pressing need, there is a scarcity of research exploring this potential mediating role. Besides, previous studies investigating the connection between maternal depression and childhood obesity have yielded mixed results. Some studies find that maternal depression is either unrelated to childhood obesity [8,11] or is even associated with lower weight in children [15,16]. However, contrasting research has shown a significant connection between maternal depression and an increased risk of childhood obesity [12,13,17]. The observed mixed findings may be related to the length of time when children are exposed to maternal depression. Research [18] indicates that chronic, rather than episodic, exposure to maternal depression has a more pronounced effect on children’s weight outcomes. Chronic maternal depression has been related to an increased risk of obesity in children, suggesting that the persistent nature of depression significantly influences parenting practices and, subsequently, child health outcomes [18]. This insight underscores the importance of considering the duration of maternal depression in studies examining its effects on child obesity. Children’s age at the time of exposure to maternal depression can have an impact on their weight outcomes. Lampard et al.’s systematic review [18] reveals that the age at which children experience maternal depression can lead to varied effects on their weight status, with the findings indicating that exposure at certain developmental stages may have distinct impacts on the risk of obesity. In addition, studies indicate that the impact of maternal depression on a child’s BMI may vary by the child’s gender. Duarte et al. [19] report that maternal depression has been associated with a higher BMI in boys, whereas its effects on girls’ BMI are less consistent. Certain research [14] shows that maternal depression may uniquely influence girls’ weight, showing no significant impact on boys indicating potential gender-specific influences of maternal mental health on childhood obesity. This complexity highlights the importance of exploring how gender influences the relationship between maternal depression and child obesity, offering the potential for a more comprehensive understanding.

This research utilized data from the Study of Early Child Care and Youth Development (SECCYD) with two aims: firstly, to investigate the impact of children’s psychosocial adjustment, specifically internalizing and externalizing behaviors, on the correlation between chronic maternal depression exposure from one month through third grade and higher BMI and overweight/obesity risk in adolescence; and secondly, to examine how the child’s gender might influence this mediation effect. We hypothesized that prolonged exposure to maternal depressive symptoms throughout early childhood would correlate with increased BMI z-scores and overweight/obesity risk at age 15, and that this association would differ based on the child’s sex. Factors such as family socioeconomic background, parental educational levels, and ethnicity were controlled for in our analysis to address potential confounders linked to maternal depression and childhood obesity [14,18].

## 2. Methods

### 2.1. Participants

The SECCYD study was initiated in 1991 at ten sites and consisted of four phases that followed children and their families from one month to adolescence in order to understand better the relationships between child development and childcare. At the beginning, this study included 1364 infants and their families, who were enlisted into the study when the infants were one month old. This study was divided into four phases: monitoring from age of 1 month to age 3 (Phase I, 1991–1994), until first grade (Phase II, 1995–1999), through sixth grade (Phase III, 2000–2004), and up to age 15 (Phase IV, 2005–2008). Initially, data collection was age-based, transitioning to a school-year schedule upon formal schooling, and reverting to age-based after seventh grade. The study experienced a 21% attrition from the original cohort by age 9, with a further 6.3% drop by age 15, leaving 1009 participants. The analysis sample in this paper included 844 participants, representing 61.9% of the initial SECCYD Phase I cohort, who provided data regarding psychosocial adjustment problems in adolescents and weight and height assessments at age 15. Initial evaluations involved contrasting the foundational attributes of participants who were accessible for analysis against those absent from the study sample, in order to assess the likelihood of selection biases. The comparative analysis showed no statistically differences in gender (*p* = 0.06), ethnicity (*p* = 0.34), children’s average reported experiences of depressive symptoms (*p* = 0.21), or the average duration of children’s exposure to maternal depressive symptoms (*p* = 0.61). Compared to the participants who were not included, the participants included in the analysis demonstrated a notably lower income-to-need ratio (4.38 compared to 5.21, *p* = 0.04), and exhibited significantly higher levels of maternal education (14.41 years compared to 13.94 years, *p* = 0.001) and older maternal age at birth (28.53 years compared to 27.23 years, *p* < 0.0001). Comprehensive information about the study can be found online (https://www.icpsr.umich.edu/web/ICPSR/series/00233/studies, accessed on 20 February 2024) and has been detailed in our prior works [5,20,21,22].

### 2.2. Measures

#### 2.2.1. Weight and Height Measurements

At the age of 15, weight and height measurements were taken following standardized procedures [5]. The calculation of BMI Z scores and the subsequent classification into overweight or obesity categories adhere to the guidelines outlined in the 2000 Centers for Disease Control and Prevention (CDC) Growth Charts, specifically designed for the pediatric and adolescent population within the US. Under these guidelines, a child’s category into the overweight or obesity category is determined by their BMI percentile, with thresholds set at exceeding the 85th percentile for being classified as overweight and above the 95th percentile for obesity classification. These criteria were applied with careful consideration of specific cutoffs for age and gender to ensure accurate and relevant categorizations [23].

#### 2.2.2. Symptoms of Maternal Depression

Evaluation of maternal depression levels was performed using the Center for Epidemiological Studies Depression Scale (CES-D), which includes 20 questions rated on a scale from 0 to 3, allowing respondents to indicate the frequency of depressive symptoms experienced over the last week. The total possible score on the CES-D can range from 0 to 60, with higher totals indicating more severe symptoms of depression [24]. This scale encompasses questions that inquire about the respondent’s feelings of sadness, hopelessness, and worthlessness, as well as questions assessing sleep patterns, appetite, and enjoyment of life. The CES-D asks specific questions such as whether the respondent has felt depressed, fearful, or lonely, among others. Throughout this study, we assessed all scoring options, considering the highest possible score of 60. To determine the extent of children’s exposure to maternal depression over time, we calculated the average score of maternal depression at different child ages (1, 6, 15, 24, 36, 54 months, and 1st, and 3rd grade) [alpha = 0.85–0.91]) [22,25] and utilized these data to assess the cumulative impact of maternal depression from one month through Grade 3.

#### 2.2.3. Externalizing and Internalizing Behaviors in Children

Evaluation of children’s internalizing and externalizing behavior issues was carried out using assessments by mothers using the Child Behavior Checklist (CBCL) [26]. The CBCL provides a comprehensive tool for parents to report on various aspects of their child’s functioning, including social skills, academic performance, emotional regulation, and specific behavior issues. It distinguishes between internalizing behaviors, such as withdrawal, somatic symptoms, and anxiety/depression, and externalizing behaviors, which include social problems, attention problems, and tendencies to delinquency and aggression. Average scores derived from the CBCL across Grades 3 to 6 provided a measure of these behavior issues over time. From grade 3 (about age 9) to grade 6 (around age 15), the inter-rater reliability for internalizing and externalizing behavior assessments was measured by correlation coefficients, which were observed to be between 0.40 and 0.47 for internalizing behaviors, and between 0.55 and 0.61 for externalizing behaviors, highlighting the consistency of these evaluations over time [20,25].

#### 2.2.4. Covariates

In our analysis, several covariates were chosen for adjustment based on their established association with child BMI and maternal mental health, as identified in literature review and previous study methodologies [21]. These included the child’s gender, the mother’s minority status—categorized into non-Hispanic Whites and other groups, maternal age at delivery, years of maternal education, the family’s income-to-need ratio by Grade 5, and the location of the study. 

### 2.3. Statistical Analysis

We described our sample using means, standard deviations, and percentages. To assess how children’s externalizing and internalizing behaviors may mediate the impact of early, prolonged maternal depression on adolescent BMI and obesity, we conducted mediation analyses using Andrew Hayes’s Process Macro within SAS software (version 9.4). We generated bias-corrected bootstrap confidence intervals from 10,000 samples, considering mediation significant if zero was not within the 95% confidence interval [27]. This bootstrapping method provides more precise results for non-normally distributed effects than traditional methods [27,28]. We adjusted for several covariates, including children’s gender, mothers’ minority status, maternal age at birth, education level, family income-to-need ratio at Grade 5, and study site location. To handle missing data, we adopted a multiple imputation strategy, conducting all mediation analyses with imputed datasets to ensure robustness (further details available upon request). We used the Markov Chain Monte Carlo (MCMC) technique in SAS’s Proc MI [29] to generate 40 imputed datasets [30,31]. These datasets were then reanalyzed to confirm the mediation effects, with SAS Proc MIANALYSIS summarizing and interpreting the findings [29]. Consistency between the imputed and original data affirmed the reliability of our results.

## 3. Result

Table 1 presented descriptive information about the study’s participants. The majority (82.23%) of mothers identified as non-Hispanic White, while the remaining (17.77%) were from a variety of ethnic minorities. At the age of 15, nearly one-third (31.04%) of the adolescents fell into the overweight or obese categories, with a notable gender disparity: 36.28% of boys versus 25.88% of girls (*p* = 0.001) were affected. Gender did not influence other variables such as maternal ethnicity, maternal age at childbirth, household income-to-needs ratio at Grade 5, adolescent BMI z-score at age 15, average long-term exposure to maternal depressive symptoms from one month to Grade 3, or assessments of internalizing and externalizing behaviors from Grade 3 to 6.

Table 2 revealed insights into the mediating role of psychosocial adjustment and how gender influenced the long-term effects of maternal depression exposure on BMI Z scores and the probability of being overweight or obese during adolescence. Utilizing bootstrapping techniques for bias correction, the study identified significant mediation by assessing the 95% confidence intervals for the exclusion of zero, providing a robust methodological approach to ascertain the mediation effects (detailed in the Methods section). In girls, the research indicated that both internalizing and externalizing behavioral problems significantly mediated the association between prolonged exposure to maternal depression and an increase in BMI Z scores (mediation effect of internalizing behavioral problems = 0.0071, 95% CI: 0.0027, 0.0125; mediation effect of externalizing behavioral problems = 0.0062, 95% CI: 0.0030, 0.0111). Similarly, these behavioral issues mediated the elevated logit (the natural logarithm of the odds) of being overweight or obese by the age of 15 (mediation effect of internalizing behavioral problems = 0.0144, 95% CI: 0.0031, 0.0288; mediation effect of externalizing behavioral problems = 0.0104, 95% CI: 0.0019, 0.0217) at age 15 years. Conversely, for boys, the investigation found no significant mediation effects on either behavioral problem.

Table 3 presented the outcomes regarding the direct influence of psychosocial adjustment on the lasting consequences of exposure to maternal depressive symptoms on BMI Z scores and the risk of overweight/obesity, segmented by gender. In boys, significant direct impacts were noted from both internalizing and externalizing behavioral problems on the increase in BMI Z scores at age 15, after prolonged exposure to maternal depressive symptoms (direct effect of internalizing behavioral problems = 0.0157, 95% CI: 0.0003, 0.0310; direct effect of externalizing behavioral problems = 0.0156, 95% CI: 0.0003, 0.0310). However, these behavioral issues did not significantly affect the natural log odds of becoming overweight or obese. In contrast, within the girls, there were no significant direct effects on BMI Z scores or the likelihood of overweight/obesity from either behavioral problem.

## 4. Discussion

This research demonstrated that behavioral problems in children acted as mediators between prolonged exposure to maternal depressive symptoms during their early years and an increase in BMI Z-score and overweight/obesity risks by late adolescence. These results align with existing studies, underscoring the negative impact of maternal depression in childhood on subsequent behavioral outcomes [32,33,34,35]. Depressed mothers display less attachment and involvement with their children compared to non-depressed mothers [35,36]. Brummelte et al. [35] report that mothers experiencing depression often engage in less sensitive and more disrupted parenting behaviors compared to their non-depressed counterparts, potentially leading to adverse developmental outcomes in their children. Lovejoy et al. [36] identify that mothers suffering from depression often exhibit diminished emotional involvement and less effective communication with their children, leading to an environment characterized by decreased positive interactions and increased negative behaviors compared to non-depressed mothers. When children receive less parental care or do not have enough time for mother-child engagement, they may develop psychosocial adjustment problems, such as internalizing and externalizing behavioral problems [32,33]. Park et al. [32] find that distinct patterns of maternal depressive symptoms are associated with higher instances of behavioral problems in children, particularly in those whose mothers’ symptoms increased over time. Mothers with increasing depressive symptoms are more likely to exhibit problem behaviors. As depressive symptoms escalate, they can impair the maternal ability to provide consistent, attentive care and engage in enriching interactions with their children, which are vital for healthy psychosocial development. The impact of maternal depression on the adjustment of children’s behavioral problems may also increase as children age [11,37]. Gjerde et al. [11] underscore that the negative impacts of maternal depression are not confined to infancy but continue to affect children’s psychosocial development into later childhood. It highlights a critical period during the preschool years when maternal depression may particularly impede children’s ability to develop adequate social and emotional coping mechanisms, thereby increasing the risk of long-term psychosocial adjustment issues. Children with internalizing and externalizing problems may be more likely to exhibit emotional eating and less active lifestyles, both of which are associated with childhood overweight [5,20,38]. Camfferman et al. [38] highlight that behavioral problems serve not only as emotional responses but also as mechanisms that may contribute to unhealthy lifestyle choices leading to overweight. 

In our study, we identified a gender-specific mediating effect of internalizing and externalizing behavioral issues, which arise from prolonged exposure to maternal depression in early childhood, on the subsequent increased risk of becoming overweight or obese and higher BMI levels in adolescence. Notably, this mediating influence was exclusively seen in girls. Therefore, it is necessary to consider gender variations when investigating the mediation effects that could influence the association between early childhood exposure to maternal depression and later elevated BMI. This gender disparity may be linked to the differential impacts of maternal depression, as evidenced by research indicating that girls might be more vulnerable to the symptoms of maternal depression [22]. Girls may internalize the effects of maternal depression more deeply, affecting their self-esteem and body image, and potentially leading to behaviors such as emotional eating or inactivity, thereby increasing obesity risk [22]. Moreover, sociocultural influences contribute to gender disparities in obesity rates. Dietary preferences, societal norms around body image, and physical activity levels differ between boys and girls, with girls often facing greater pressure to adhere to thinness ideals [39]. These pressures can lead to different eating behaviors and concerns about weight between genders, potentially impacting obesity rates. Additionally, parental attitudes towards their children’s weight can differ by gender, with some studies indicating that parents may express more concern about their girls’ weight status compared to their boys [39,40]. In our study, boys were found to have a direct increase in BMI or obesity/overweight risk in response to maternal depression. This gender-specific effect is supported by research indicating that boys and girls are affected differently by maternal mental health and parenting practices due to varied sociocultural expectations and biological factors. Boys, who may receive less supervision from depressed mothers, are less subject to societal pressures regarding physical appearance compared to girls, possibly leading to less regulated eating habits and physical inactivity [39]. They may be at direct risk of weight gain due to less parental supervision or care resulting from maternal depression [22,40].

Our research highlights how maternal depression distinctly affects boys’ and girls’ overweight/obesity risks, underlining the need for early psychosocial intervention. We demonstrate that both internalizing and externalizing behaviors significantly impact increased weight, particularly in girls, revealing the complexity of these relationships and advocating for gender-tailored preventive measures. This finding stresses the importance of addressing both behavioral and physical health in children from a young age. Therefore, designing gender-sensitive interventions—targeting boys’ direct risks and girls’ specific challenges related to maternal interaction—is crucial. Such strategies should carefully address the complex influence of maternal depression on children, especially focusing on girls’ unique susceptibilities. This insight is crucial for creating holistic interventions that cater to the psychological and physical health of children impacted by maternal depression.

This study has several limitations. First, the generalizability of the findings may be limited the predominance of non-Hispanic White participants in our sample analysis. Future research should aim for a more diverse participant base to enhance the generalizability of findings across different racial and socioeconomic groups. Second, there is a possible risk of self-selection bias within the study. The sample analyzed represents only 61.9% of the initial SECCYD sample size. Those participants who continued in the study tended to be more educated and less in need [22]. As an effort of the sensitivity analysis, we did multiple imputation approach to address missing data and replicated mediation analysis (results were not presented in the article but are available on request). Consistent results were observed when comparing imputed data and raw data analysis, which highlights the robustness of our study findings. Third, a methodological limitation in our study was the accurate determination of the duration of children’s exposure to maternal depressive symptoms, due to the difficulty in identifying the exact beginning and end of depressive episodes. Longitudinal studies that track the onset and progression of maternal depressive symptoms, alongside detailed assessments of children’s health outcomes, would provide richer data. It would allow for a more comprehensive understanding of how the timing, severity, and duration of maternal depression interact with various child development markers.

## 5. Conclusions

Our study found a significant association between prolonged maternal depression from early childhood to significant behavioral problems and subsequent increases in BMI and overweight or obesity risks in adolescence, with this relationship notably present in girls. This discovery underscores the importance of incorporating gender considerations into obesity intervention strategies. In the future, studies should explore other factors that may influence childhood obesity. Exploring the role of paternal mental health and its interaction with maternal depression and child outcomes may provide a more comprehensive view of family dynamics affecting child development. Other mediation and moderation effects, such as family cultural and religious backgrounds, children’s eating habits, and maternal BMI, should also be researched to improve childhood obesity control.

## Figures and Tables

**Table 1:** Demographic and Behavioral Characteristics of the Sample, Stratified by Gender.

	Whole Sample	Boys	Girls	*p*-Value
Maternal Ethnic Distribution, n (%)				0.66
Non-Hispanic Whites	694 (82.23%)	347 (82.82)	347 (81.65)	
All except non-Hispanic Whites	150 (17.77%)	72 (17.18)	78 (18.35)	
Mother’s Delivery Age, mean (SD)	28.53 (5.56)	28.27 (5.69)	28.80 (5.42)	0.17
Average Maternal Educational Attainment (Years), mean (SD)	16.12 (2.12)	16.03 (2.16)	16.21 (2.08)	0.2
Family Income-to-Need ratio at Grade 5, mean (SD)	3.78 (2.74)	3.68 (2.64)	3.89 (2.83)	0.26
Body Weight Status at age 15				0.001
Normal/underweight, n (%)	582 (68.96)	267 (63.72)	315 (74.12)	
Overweight/Obesity, n (%)	262 (31.04)	152 (36.28)	110 (25.88)	
Adolescent BMI Z-score at Age 15, mean (SD)	0.57 (0.99)	0.63 (1.08)	0.52 (0.91)	0.12
Average Score of Maternal Depressive Symptom Exposure from 1 Month to Grade 3, mean (SD)	9.80 (6.56)	9.67 (6.56)	9.93 (6.56)	0.58
Average Psychosocial Adjustment Score, Grades 3 to 6, mean (SD)				
Internalizing Behaviors	48.33 (8.68)	48.31 (9.12)	48.35 (8.24)	0.95
Externalizing Behaviors	46.63 (8.91)	46.45 (8.54)	46.81 (9.27)	0.55

SD, standard deviation.

**Table 2:** Mediation Effects of Behavioral Problems on BMI Z Scores and Overweight/Obesity Risks by Gender.

	BMI Z Score as Outcome			Overweight/Obesity as Outcome		
	Whole Sample	Boys	Girls	Whole Sample	Boys	Girls
Internalizing behavioral problems	0.0038 (0.0005, 0.0073) *	0.0011 (−0.0036, 0.0059)	0.0071 (0.0027, 0.0125) *	0.0054 (−0.0022, 0.0133)	−0.0008 (−0.0115, 0.0091)	0.0144 (0.0031, 0.0288) *
Externalizing behavioral problems	0.0040 (0.0015, 0.0070) *	0.0018 (−0.0016, 0.0059)	0.0062 (0.0030, 0.0111) *	0.0057 (−0.0003, 0.0125)	0.0015 (−0.0069, 0.0104)	0.0104 (0.0019, 0.0217) *

BMI, body mass index. * Mediation effects were deemed significant when zero was not present within the 95% confidence intervals, derived from bias-corrected bootstrap estimations in the mediation analysis via linear regression.

**Table 3:** Direct Effects of Behavioral Problems on BMI Z Scores and Overweight/Obesity Risks by Gender.

	BMI Z Score as Outcome			Overweight/Obesity as Outcome		
	Whole Sample	Boys	Girls	Whole Sample	Boys	Girls
Internalizing behavioral problems	0.0069 (−0.0043, 0.0181)	0.0157 (0.0003, 0.0310) *	−0.0023 (−0.0176, 0.0129)	0.0132 (−0.0121, 0.0384)	0.0230 (−0.0109, 0.0570)	−0.0002 (−0.0359, 0.0354)
Externalizing behavioral problems	0.0066 (−0.0044, 0.0177)	0.0156 (0.0003, 0.0310) *	−0.0018 (−0.0168, 0.0131)	0.0128 (−0.0122, 0.0378)	0.0222 (−0.0116, 0.0560)	0.0025 (−0.0328, 0.03787)

BMI, body mass index. * Mediation effects were deemed significant when zero was not present within the 95% confidence intervals, derived from bias-corrected bootstrap estimations in the mediation analysis via linear regression.

## Data Availability

The data presented in this study are available in article.
